# Enhanced Light Emission in MoSe_2_–WSe_2_ Lateral Heterostructures in the Electron–Hole Plasma Regime

**DOI:** 10.1021/acs.jpclett.5c02100

**Published:** 2025-08-05

**Authors:** Frederico B. Sousa, Bárbara A. L. Ferreira, Suman Kumar Chakraborty, Luiz C. Carvalho, Alisson R. Cadore, Biswajeet Nayak, Purbasha Ray, Simone S. Alexandre, Prasana K. Sahoo, Ricardo W. Nunes, Leandro M. Malard

**Affiliations:** † Departamento de Física, 28114 Universidade Federal de Minas Gerais, Belo Horizonte, Minas Gerais 30123-970, Brazil; ‡ Materials Science Centre, 30133 Indian Institute of Technology Kharagpur, Kharagpur, West Bengal 721302, India; ¶ Brazilian Nanotechnology National Laboratory (LNNano), 215006 Brazilian Center for Research in Energy and Materials (CNPEM), Campinas, São Paulo 13083-100, Brazil

## Abstract

Two-dimensional (2D) transition metal dichalcogenide (TMD) semiconductors exhibit interesting many-body effects even above room temperature due to their strong electron–hole interactions. For instance, low excitation densities lead to the well investigated exciton formation in these 2D TMDs and their heterostructures. The confinement of the moiré excitons and the delocalization of the interlayer excitons are among the novel excitonic phenomena presented by TMD-based heterostructures. However, the high excitation density responses of these 2D semiconductors and their heterostructures still lack solid understanding. In this work, we investigate the electron–hole plasma photoluminescence generated by high excitation densities in 2D MoSe_2_–WSe_2_ lateral heterostructures. Photoluminescence mapping and spectroscopy measurements at high pumping regimes reveal an enhanced light emission at the lateral heterojunctions. *Ab initio* calculations for a MoSe_2_–WSe_2_ lateral heterostructure with an alloyed interface of approximately the size of the heterojunctions of the experimental samples show good agreement with the experimental data. Additionally, the theoretical results provide an explanation for the observed enhancement of the photoluminescence at the heterojunctions and for the role of interfacial alloying in increasing the overlap of electron and hole wave functions at the interface. These observations reveal the localized character of the optical effects at heterojunctions of lateral TMD-based heterostructures under high excitation densities.

## Graphical abstract

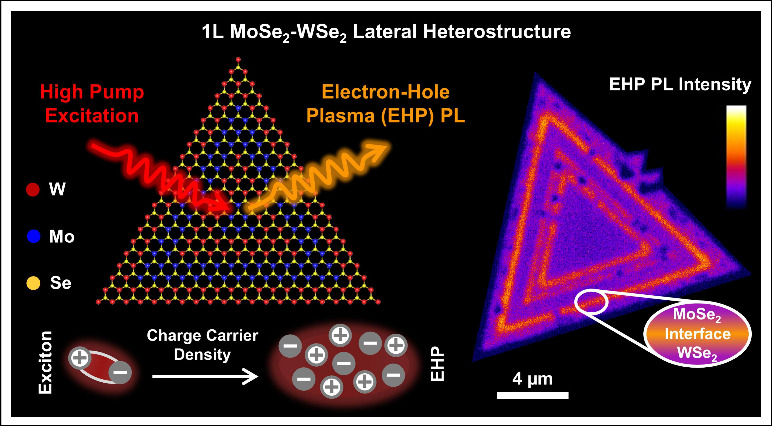

Two-dimensional (2D) van der Waals heterostructures of transition metal dichalcogenides (TMDs) are an ideal platform for the investigation of novel fundamental physical phenomena and the development of innovative technologies.^1–3^ TMD vertical heterostructures, for example, present notable excitonic effects such as the formation of interlayer and moiré excitons,^4–11^ while in-plane charge transfer excitons and trions are observed in TMD lateral heterojunctions.^12,13^ However, unlike the highly explored excitonic physics, high-excitation-density regimes in TMD heterostructures still lack careful investigations,^14^ especially for lateral heterostructures.

The high binding energy of excitons in TMD monolayers is associated with their reduced dielectric screening.^15^ However, when high charge carrier densities are generated in these materials, an enhanced electronic screening competes with the Coulomb interaction between electrons and holes. From a threshold carrier density, excitons are dissociated into a plasma of electrons and holes,^16–22^ as illustrated in Figure 1a, a process referred to as Mott transition. While excitons show sharp energy levels that result in a narrow PL peak when recombining, the spectral line width of the electron–hole plasma (EHP) light emission depends on the charge carrier density.^17,22^ Under increasing densities above the Mott threshold, the electronic thermalization induces the occupation of higher energy states and broadens the EHP PL.^17,22^ This light emission tunability, in addition to further particularities of the EHP regime in 2D TMDs, offers great opportunities for distinctive applications in optoelectronic and photonic devices.^16,22^ Particularly, this EHP formation with the PL broadening signature has also been reported for a MoSe_2_/WSe_2_ vertical heterostructure at low temperature, in which the interlayer excitons were dissociated into electron and hole plasmas localized in separate layers.^23^ Moreover, low-temperature pump–probe measurements revealed a fast expansion of the EHP also in a MoSe_2_–WSe_2_ vertical heterostructure.^24^ On the other hand, the heterojunctions of TMD lateral heterostructures still demand investigations at these high pump fluences.^14^ Such a study holds great promise, for example, in uncovering lateral interlayer phenomena at the regime of ionized electrons and holes. Hence, the investigation of the unexplored optical responses of 2D TMD lateral heterojunctions at high excitation densities is valuable for both fundamental and technological points of view.^14^ Additionally, it paves the way for the research of other types of 2D lateral heterostructures—e.g., graphene-TMD^25^ or graphene-hBN^26,27^ —at these high pump fluences, since graphene exhibits a finite EHP PL even being a gapless material.^28,29^

**Figure 1:**
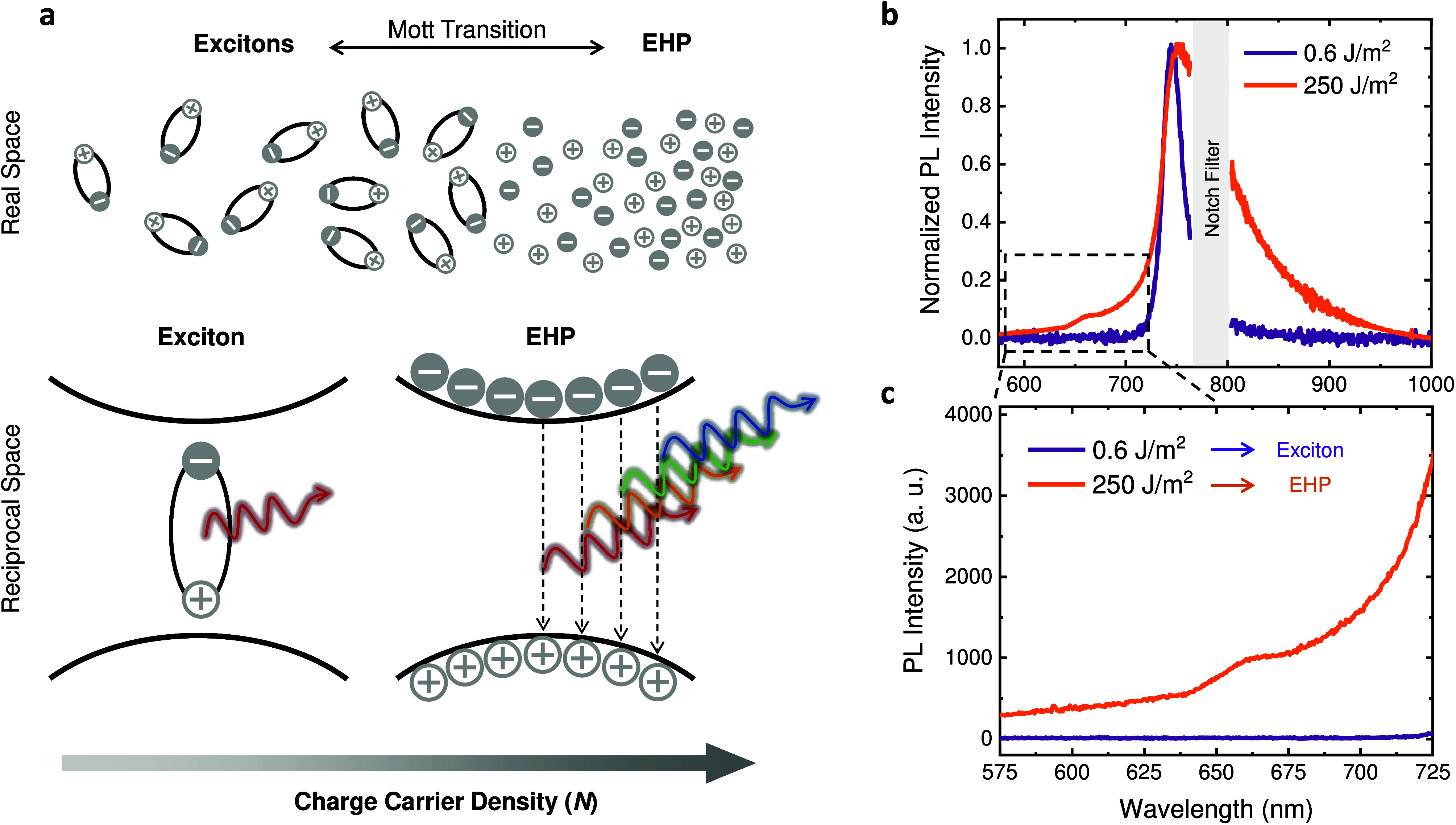
(a) Exciton and EHP illustration at the real and reciprocal spaces. (b) Normalized PL spectra of a monolayer WSe_2_ acquired with 0.6 J/m^2^ and 250 J/m^2^ pump fluences. The low excitation spectrum shows the narrow exciton PL peak, while the high excitation one displays the broadened EHP emission. The PL spectra were obtained at a 785 nm excitation and with a 785 nm notch filter, which is the reason for the gap in the lines. (c) Exciton and EHP PL spectra from the high energy region highlighted in (b).

In this work, we report PL mapping measurements at densities above the Mott threshold in monolayer (1L) and bilayer (2L) MoSe_2_–WSe_2_ lateral heterostructures. A sub-bandgap pulsed excitation was used to generate charge carrier densities of up to 10^15^ cm^–2^, which resulted in a PL broadening from the near-infrared (NIR) to the visible spectral range. A noticeable enhancement of EHP light emission was observed at the heterojunctions for a range of excitation energies, which was also detected in PL spectroscopy measurements and points to a contribution from both domains in the charge-carrier radiative recombination at the interface. To confirm the interfacial character of the observed enhancement of the PL signal, we performed Kohn–Sham Density-Functional-Theory (KS-DFT)^30,31^ first principle calculations for a 2D MoSe_2_–WSe_2_ lateral heterostructure with a diffuse alloyed interface. Using this methodology, we computed the absorption spectrum of the 2D heterostructure, which revealed that the charge density for the initial and final electronic states of the calculated absorption resonances presents an overlap at the alloyed heterojunction, corroborating the strong interfacial character of the transition. Our calculations indicate that alloying enhances the overlap between electron and hole wave functions at the interface, resulting in the observed increase of the PL signal at the heterojunctions for the EHP regime probed in our experiments. Therefore, our results unveil a singular optical response of a TMD heterojunction under high excitation densities, highlighting its potential for interlayer effects at the ionized electrons and holes regime. Besides, we argue that the EHP regime is the ideal scenario for the application of the interacting-electron-gas-based generalized-gradient approximation (PBE-GGA) to the exchange and correlation effects that we employ in our calculations.

Recently, we have shown that a sub-bandgap pulsed optical excitation induces charge densities above the Mott threshold in diselenide TMDs,^22^ leading to a broad EHP emission ranging from 500 to 950 nm. This broadening is presented in the exciton and EHP normalized PL spectra of a WSe_2_ monolayer displayed in Figure 1b. In particular, for energies above 1.71 eV (Figure 1c), i.e., below 725 nm, a substantial EHP emission contrasts with a negligible exciton PL. Additionally, Figure S1 in the Supporting Information (SI) shows the power dependence of the WSe_2_ monolayer PL, evidencing the Mott transition at approximately 10 J/m^2^. These measurements were performed at a 785 nm excitation from a picosecond laser operating at 80 MHz and using a 785 nm notch filter before the detector to block the laser. The finite PL signal observed for this sub-bandgap excitation is attributed to a multiphonon upconversion process.^32^ Furthermore, the EHP PL broadening at the lower energy range of the spectrum is due to a band gap renormalization at high excitation densities.^16,22^ In this work, we employed a similar sub-bandgap pulsed excitation above the Mott threshold to generate and investigate the EHP emission in TMD lateral heterojunctions, as described in the SI (Section S1).

The first heterostructure sample investigated in this work is a three-junction 1L-MoSe_2_–WSe_2_ lateral heterostructure synthesized by the one-pot method^33^ (see Section S1 in the SI for the sample fabrication details). This method provides heterojunctions with an abrupt transition of a few nm,^33^ which varies when the TMD order is changed. While the transition from the WSe_2_ to the MoSe_2_ domain (in the growth direction) is extremely sharp (∼1 nm), we observe the formation of an alloyed interface approximately 6 nm wide in the reverse transition.^33^ Figure 2a shows the schematic representation of the three-junction 1L-MoSe_2_–WSe_2_ lateral heterostructure. Optical images of the lateral heterostructures studied in this work are presented in Figure S2 in the SI, showing the alternation of MoSe_2_ and WSe_2_ domains from the center to the edge, respectively. PL and Raman characterizations of the samples are displayed in SI Figure S3, confirming the 1L nature of both materials.

**Figure 2:**
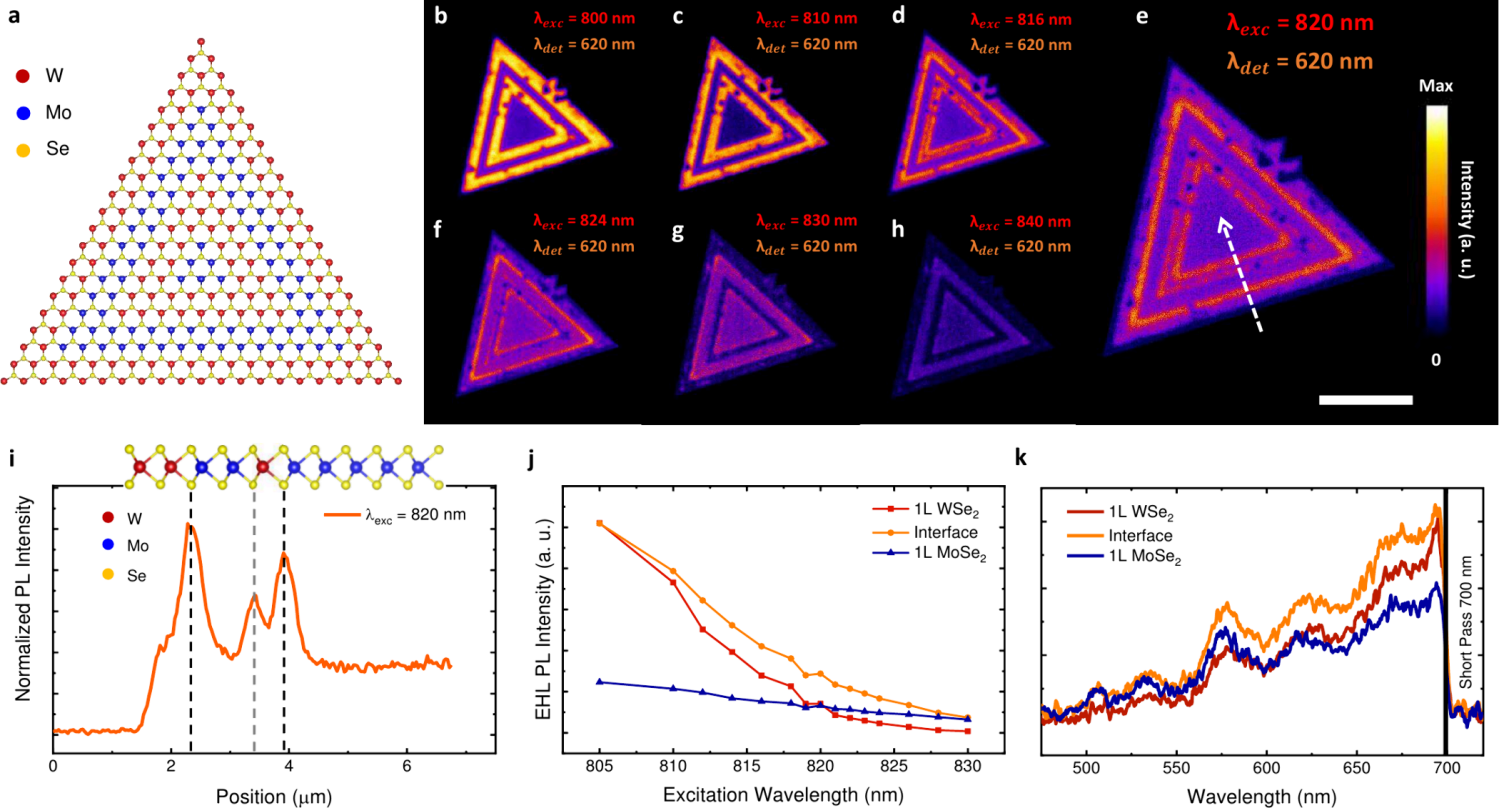
(a) Schematic representation of a three-junction 1L-MoSe_2_–WSe_2_ lateral heterostructure. (b–h) EHP PL mapping of a 1L-MoSe_2_–WSe_2_ lateral heterostructure for 800 nm (b), 810 nm (c), 816 nm (d), 820 nm (e), 824 nm (f), 830 nm (g), and 840 nm (h) excitation wavelengths and 900 J/m^2^ pump fluence. The EHP PL signal was collected by a PMT with a 620/60 nm band-pass filter. Scale bar relative to (e): 5 μm. (i) EHP PL intensity profile plot in the direction shown in the arrow of (e) for a 820 nm excitation wavelength. The black and gray vertical dashed lines indicate the alloyed and sharp heterojunction positions, respectively. (j) EHP PL intensity as a function of the excitation wavelength of a 1L-MoSe_2_–WSe_2_ lateral heterostructure. The PL intensities of the WSe_2_, MoSe_2_, and interface regions clearly show the excitation wavelength dependence features observed in the PL images of (a–g). (k) EHP PL spectra of a 1L-MoSe_2_–WSe_2_ lateral heterostructure with an 805 nm excitation, a 500 J/m^2^ pump fluence and a 700 nm short pass filter placed in front of the spectrometer. The spectra obtained for WSe_2_, MoSe_2_, and interface regions confirm the enhanced emission at the heterojunction. The oscillations along the spectra are attributed to artifacts from optical elements.

Figures 2b-h show EHP PL images of the MoSe_2_–WSe_2_ lateral heterostructure for distinct pulsed excitation wavelengths (λ_*exc*_) and a pump fluence of 900 J/m^2^. A 620/60 nm (λ_*det*_) band-pass filter was placed in front of the photomultiplier tube (PMT) to collect only the EHP signal and block the excitonic emission, as demonstrated in Figures 1b,c). The high excitation power used here is capable of generating charge densities as high as 10^15^ cm^–2^ without damaging the sample, well above the Mott threshold^17,21,22,34^ (see Section S2 in the SI for the charge carrier density calculation details). This is possible because of the sub-band gap excitation and fast pixel dwell time (few μs) used for the EHP PL images of Figures 2b-h. Moreover, the nonlinear power dependence of the EHP PL intensity for MoSe_2_, WSe_2_, and interface regions further confirms the Mott transition and thus the generated EHP regime, as shown in SI Figure S4. An EHP PL intensity dependence on the excitation wavelength is seen in Figures 2b-h. In addition to an EHP PL intensity decrease in the whole sample with increasing excitation wavelength, the PL intensity from MoSe_2_ domains relatively increases compared to WSe_2_ domains. This intensity inversion between these materials is due to the lower bandgap energy of the MoSe_2_. Notably, an enhanced EHP PL intensity at the heterojunctions can also be observed in Figures 2c-g. The signal at heterojunctions is stronger when the PL intensities of both domains are similar, reaching an enhancement factor greater than 2 for 820 nm excitation wavelength, as shown in the intensity profile plot of Figure 2i. Moreover, the intensity profile plot also reveals different enhancements at distinct heterojunctions. The strongest enhancement is observed at the interfaces in which there is an alloy formation (from MoSe_2_ to WSe_2_), while the sharp heterojunction (from WSe_2_ to MoSe_2_) shows a weaker enhancement. It is worth noting that this EHP PL enhancement at heterojunctions was observed for several 1L-MoSe_2_–WSe_2_ lateral heterostructures pumped by distinct incident powers and with emissions detected over different energy ranges, as displayed in SI Figures S5–S7.

To better visualize the excitation wavelength dependence of the 1L-MoSe_2_–WSe_2_ lateral heterostructure EHP PL, we extracted the intensities from both materials and at the interface for the distinct measured excitation wavelengths (Figure 2j). The intensities were extracted from the EHP PL images displayed in Figures 2b-h by taking the mean value over the MoSe_2_, WSe_2_, and heterojunction areas. From low to higher excitation wavelengths, the EHP PL intensities from MoSe_2_, WSe_2_, and heterojunction decrease monotonically. The general behavior can be understood in terms of the sub-bandgap excitation, in which the multiphonon upconversion process responsible for generating excited carriers becomes weaker for increasing excitation wavelengths.^32^

We have also checked the dependence of the emitted EHP PL on the excitation laser polarization, as shown in SI Figure S8. Contrary to what was reported for the second-harmonic generation enhancement at the heterojunctions of a 1L-MoSe_2_–WSe_2_ lateral heterostructure,^35^ the EHP PL emission is not polarized and thus our observed enhancement at the heterojunctions cannot be explained by a coherent interference between each domain signal. Therefore, the EHP PL enhancement suggests that charge carriers from both domains contribute to the radiative recombination at the heterojunction, since it is stronger at the alloyed interface and when the MoSe_2_ and WSe_2_ emissions are similar. In particular, this agrees with a previous work that reported electron and hole plasmas localized in separate layers in a MoSe_2_/WSe_2_ vertical heterostructure,^23^ resulting in an interlayer EHP emission. To further explore this phenomenon, we have also obtained EHP PL spectra at MoSe_2_, WSe_2_, and heterojunction regions, as shown in Figure 2k. For these experiments, a 700 nm short pass was placed in front of the spectrometer to block the laser and exciton signals, and thus collect only the high-energy tail of the EHP emission, as demonstrated in Figures 1b,c). Since the spectroscopy experiments take much longer (15 s integration time), the pump fluence is lowered to 400 J/m^2^ to avoid sample damage. For this fluence, the excitation wavelength in which the enhancement at the heterojunction is more evident is 805 nm (see SI Figures S5–S7). It is possible to observe that the three regions have a broad spectrum ranging down to 500 nm, and that there is indeed a stronger signal from the heterojunction for this wavelength range, confirming the features observed in the imaging measurements. Note, however, that the enhancement at the interface is considerably lower than in the imaging experiments due to the reduced excitation fluence, which is expected since the EHP PL has a nonlinear power dependence. Finally, it is important to mention that the oscillations present in the EHP spectra from all three regions are artifacts that come from the optical elements of the experimental setup.

To gain deeper insights into the experimental results, we performed KS-DFT calculations for the absorption spectrum of a MoSe_2_–WSe_2_ 2D periodic heterostructure, shown in Figure 3a (see Section S1 in the SI for theoretical calculation details). The lateral size of the calculated MoS_2_ and WSe_2_ domains is 2.7 nm, and we consider a diffuse alloyed interface extending over ∼ 3.3 nm, i.e., on the scale of the alloyed interfaces of the samples in our study, as depicted in Figure 3b. Figure 3a shows the theoretical absorption spectrum for the alloyed interface, which exhibits multiple resonances associated with different optical transitions that are denoted in the DOS and PDOS graphs of SI Figure S9. For each of these transitions, we calculated the charge density isosurfaces for the initial and final states. Figure 3c shows representative isosurfaces for the transitions at 2.08 eV (596 nm) and 2.32 eV (534 nm), while SI Figure S9 displays the isosurfaces for several other transitions. The overlap between the charge densities of the initial and final states in the heterojunction for all of these transitions indicates the interfacial nature of the transition in the 2D heterostructure. Therefore, this localization of the carrier wave functions results in a higher EHP density at the alloyed interfaces, consistent with the enhanced and broadened EHP PL at the heterojunctions.

**Figure 3:**
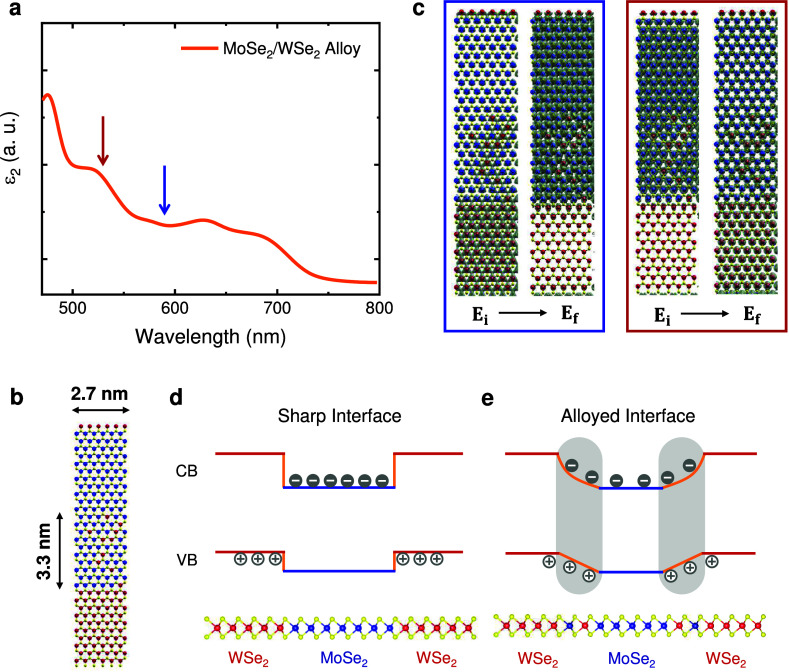
(a) DFT-calculated imaginary part of the dielectric function (ϵ_2_) for an alloyed 1L-MoSe_2_–WSe_2_ lateral heterostructure shown in (b). (c,d) Isosurfaces of charge density for the initial and final states of the transitions at 2.08 eV (596 nm) and 2.32 eV (534 nm). These transitions are indicated by the blue and red arrows in (a), and denote the isosurfaces inside the blue and red rectangles in (c), respectively. (d) Type-II band alignment of the 2D MoSe_2_–WSe_2_ heterostructure with a sharp interface trapping electrons and holes in different regions of the heterostructure. (e) Realistic picture of the potential wells at the interface with bowing in the conduction band and the linear interpolation of the valence band levels along the alloyed interface. Low-energy electron and hole states extend over the interface and show slower exponential decay into the barrier regions due to the energy-dependent barriers associated with the profile of conduction and valence band wells, leading to enhanced overlap of electron and hole wave functions for low-energy transitions.

To further illustrate this interfacial PL phenomenon, Figures 3d,e show the type-II band alignment of valence and conduction bands in the monolayer MoSe_2_–WSe_2_ lateral heterostructure, as obtained in previous theoretical calculations of Mo_*x*_Se_2_–W_1–*x*_Se_2_ alloys.^36,37^ These calculations show that, given the nature of the electronic states at the top of the valence band and bottom of conduction bands in MoSe_2_ and WSe_2_ monolayers, the top of the valence band behaves as a linear interpolation between the values for the two end systems along the interfacial alloy, while bowing is observed in the bottom of the conduction band. Figure 3d shows a schematic view of the confinement of low-energy electron and hole states in different regions of the heterostructure for the theoretical limit of a completely sharp interface. In contrast, Figure 3e presents a realistic scenario of the interfacial potential wells, where both electron and hole states extend over the interface and exhibit slower exponential decays due to the gradual energy barriers. An increase in the overlap between low-energy electron and hole states is thus obtained, which explains the enhancement of the EHP PL intensities at the interface. Besides, the effect is stronger for larger interfacial regions, in agreement with our experimental results that show higher EHP PL signals at the alloyed interface.

This uncovering of localized interfacial effects in 2D lateral heterostructures at high charge carrier densities also strengthens the importance of future works to directly probe the electronic structure of these materials. While angle-resolved photoemission spectroscopy (ARPES) has been recently used for such a study in vertical heterostructures,^38–40^ the advancements in the micro-ARPES technique will enable this investigation for 2D lateral heterojunctions as well.

Regarding our theoretical results, we emphasize that the EHP regime of transitions that we address in our experiments is an ideal scenario for the KS-DFT calculations we employ. The very high density of the EHP, with a Wigner-Seitz parameter of *r*_*s*_ = 3.4, is well within the homogeneous phase of the interacting electron (hole) gas, and very far from the threshold *r*_*s*_^*thr*^ = 37 ^41^ for transition to a Wigner crystal, where Coulomb-interaction effects become dominant. At *r*_*s*_ = 3.4, we have a highly degenerate and highly screened 2D EHP dominated by kinetic energy effects. This is the scenario where the KS-DFT mean-field approach to exchange and correlation effects, within the PBE-GGA approximation, provides a very accurate description of the system. In the context of our MoSe_2_–WSe_2_ heterostructure, the preponderance of interaction effects would lead to the Mott threshold for exciton formation at *r*_*s*_ ∼ *r*_*s*_^*trh*^.

Finally, to study the influence of the sample thickness in the EHP PL emission, we performed similar PL mapping measurements for 2L-MoSe_2_–WSe_2_ lateral heterostructure. The sample optical characterization is presented in Supporting Figure S10. Figures 4a-i show EHP PL images of a 2L-MoSe_2_–WSe_2_ lateral heterostructure for distinct excitation wavelengths, an incident pump fluence of 500 J/m^2^, and a 620/60 nm bandpass filter placed in front of the PMT. An EHP PL enhancement at the heterojunctions can also be observed for this 2L lateral heterostructure, as displayed in Figures 4d-f. Besides, the enhancement at the alloyed interfaces is stronger as well. However, in contrast to the monotonic decrease in the EHP PL intensity by decreasing the excitation wavelength presented by the 1L lateral heterostructure (Figure 2), a different excitation wavelength dependence is noticed for the 2L-MoSe_2_–WSe_2_ lateral heterostructure. Figure 4j shows the EHP PL intensity graph as a function of the excitation wavelength for 2L-WSe_2_ and MoSe_2_ regions and their heterojunction. A resonant response at 820 and 840 nm for WSe_2_ and MoSe_2_ domains can be noted, respectively. These resonances endorse the relation between the EHP PL intensity and broadening with the material absorption. Moreover, as well as for the 1L lateral heterostructure, the enhanced EHP PL emission at the 2L heterojunction happens for the excitation wavelengths in which the intensities from the individual domains are similar. Hence, our experimental results reveal that atomically thin MoSe_2_–WSe_2_ lateral interfaces present a remarkable luminescence emission at high excitation densities. Since this emission can be tuned by the excitation wavelength, incident power, sample thickness, and interface width, our results evidence a great potential for these heterojunctions for tunable emitting devices at high carrier density regimes. Additionally, the interfacial nature of the EHP recombination at the heterojunction contributes to understanding the fundamental dynamics and interlayer effects of ionized charge carriers in 2D TMD lateral heterostructures.

**Figure 4:**
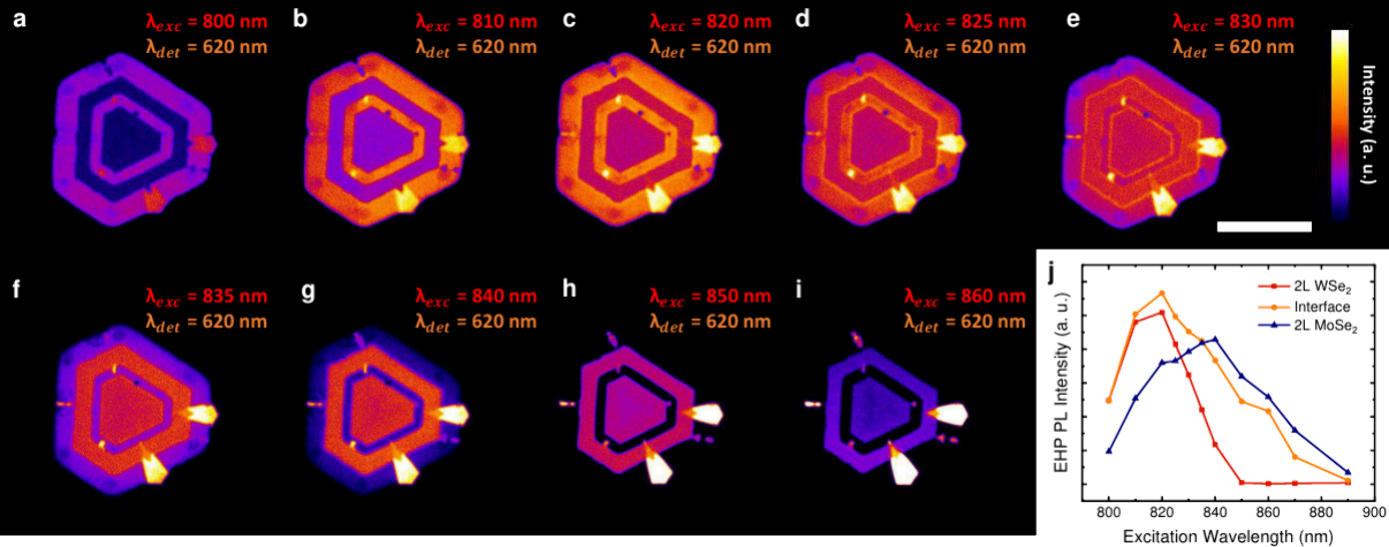
(a–i) EHP PL mapping of a 2L-MoSe_2_–WSe_2_ lateral heterostructure for 800 nm (a), 810 nm (b), 820 nm (c), 825 nm (d), 830 nm (e), 835 nm (f), 840 nm (g), 850 nm (h), and 860 nm (i) excitation wavelengths and a 500 J/m^2^ pump fluence. The EHP PL signal was collected by a PMT with a 620/60 nm bandpass filter. (j) EHP PL intensity as a function of the excitation wavelength of a 2L-MoSe_2_–WSe_2_ lateral heterostructure. The PL intensities of the WSe_2_, MoSe_2_, and interface regions show the excitation wavelength dependence features observed in the PL images of (a–i). Scale bar relative to (e): 20 μm.

In summary, EHP PL imaging measurements revealed an enhanced intensity at heterojunctions of 1L- and 2L-MoSe_2_–WSe_2_ lateral heterostructures. These results were corroborated by EHP PL spectroscopy measurements, which also showed a more intense signal at the lateral interfaces. KS-DFT calculations for the absorption spectrum of a 2D MoSe_2_–WSe_2_ heterostructure allowed us to examine the charge densities for the initial and final states of different optical transitions, which pointed to the overlap of electron and hole wave functions at the interface. Besides, our calculations indicate that interfacial alloying leads to an enhancement of this overlap between low-energy electron and hole states at the heterojunctions. This localization means a higher carrier density at the lateral interfaces and hence an enhanced and broadened EHP emission, underlying the agreement between our measured and calculated results. Therefore, our work reveals the singular, controllable, and localized optical response of TMD lateral heterojunctions under high excitation densities.

## Supplementary Material



## Notes

Experimental and theoretical methods, charge carrier densities calculation, PL power dependence, optical images, Raman and PL characterization, additional EHP PL imaging, polarization-resolved EHP PL imaging, and charge density isosurfaces for the initial and final states of different optical transitions (PDF)
