# Supplementary material for: Enhanced Light Emission in MoSe2–WSe2 Lateral Heterostructures in the Electron–Hole Plasma Regime
Source: J Phys Chem Lett. 2025 Aug 5;16(32):8227–33. doi: 10.1021/acs.jpclett.5c02100 (PMC12359105; doi:10.1021/acs.jpclett.5c02100)
Supplement: Supplementary file 1 [file jz5c02100_si_001.pdf]

Supporting Information for Enhanced Light Emission in $MoSe_2$-WSe_2 Lateral Heterostructures in the Electron-Hole Plasma Regime

Frederico B. Sousa[1], Bárbara A. L. Ferreira[1], Suman Kumar Chakraborty[2], Luiz C. Carvalho[1], Alisson R. Cadore[3], Biswajeet Nayak[2], Purbasha Ray[2], Simone S. Alexandre[1], Prasana K. Sahoo[3], Ricardo W. Nunes[1], and Leandro M. Malard[1]*

[1]Departamento de Física, Universidade Federal de Minas Gerais, Belo Horizonte, Minas Gerais 30123-970, Brazil

[2]Materials Science Centre, Indian Institute of Technology Kharagpur, Kharagpur, West Bengal, 721302, India

[3]Brazilian Nanotechnology National Laboratory (LNNano), Brazilian Center for Research in Energy and Materials (CNPEM), Campinas, SP 13083-100, Brazil

*lmalard@fisica.ufmg.br

This Supporting Information includes:

• Section S1. Experimental and theoretical methods.

• Section S2. Charge carrier densities calculation.

• Figure S1. PL power dependence for 1L-WSe_2.

• Figure S2. 1L- and 2L-$MoSe_2$-WSe_2 lateral heterostructure optical images.

• Figure S3. Raman and PL characterization for 1L-$MoSe_2$-WSe_2 lateral heterostructure.

• Figure S4. PL power dependence for 1L-$MoSe_2$-WSe_2 lateral heterostructure.

• Figure S5. EHP PL imaging for 1L-$MoSe_2$-WSe_2 lateral heterostructure (Flake 1).

• Figure S6. EHP PL imaging for 1L-$MoSe_2$-WSe_2 lateral heterostructure (Flake 2).

• Figure S7. EHP PL imaging for 1L-$MoSe_2$-WSe_2 lateral heterostructure (Flake 3).

• Figure S8. EHP PL polarized measurement for 1L-$MoSe_2$-WSe_2 lateral heterostructure.

• Figure S9. Charge density isosurfaces for the initial and final states of different optical transitions.

• Figure S10. Raman and PL characterization for 2L-$MoSe_2$-WSe_2 lateral heterostructure.

Section S1. Experimental and theoretical methods

Sample Preparation

This study employed a water-assisted chemical vapor deposition (CVD) method to synthesize 2D $MoSe_2$-WSe_2 lateral heterostructures under atmospheric pressure conditions, using bulk $MoSe_2$ and WSe_2 as CVD precursors[1–3]. The experimental setup consisted of a 1-inch diameter quartz tube placed horizontally within a two-zone CVD furnace. High-purity alumina boats held the precursor powders containing $MoSe_2$ and WSe_2 inside this tube. The substrates used for TMD deposition were Si with 285 nm thick SiO_2, which were methodically cleaned using acetone, isopropanol, and deionized water. Substrates were positioned downstream in the quartz tube, maintaining a temperature range of 800-750 °C. They were located at a distance of 6-10 cm from the solid precursor sources situated at 1050 °C. The furnace temperature was slowly raised from room temperature to 1050 °C for 50 minutes. This temperature increase was facilitated by a constant flow of nitrogen (N_2) at 200 standard cubic centimeters per minute (sccm). Once the furnace temperature exceeded 1000 °C, we inserted the solid precursor powders and substrates into their designated positions by sliding the quartz tube into the furnace. Simultaneously, we introduced controlled amounts of water vapor into the system by diverting the N_2 flow through a bubbler containing 2 ml of deionized water at room temperature. To facilitate the transition from Mo-rich to W-rich compounds and the fabrication of lateral heterostructures, we swiftly replaced the N_2+H_2O vapor flux with a mixture of argon (Ar) and 5% hydrogen (H_2) at a flow rate of 200 sccm. The size of domains within the lateral heterostructures was adjusted by varying the deposition time, whereas the layer number was controlled by adjusting the substrate temperature and amount of precursors[2,3]. After achieving the desired heterostructure sequences, we terminated the synthesis process

by sliding the quartz tube containing the precursor powders and substrates into a cooler zone. Throughout this cooling phase, a constant flow of Ar+H_2 (5%) at 200 sccm was maintained until the system reached room temperature. The growth of multi-junction TMD domains was achieved by sequentially switching the carrier gases: N_2+H_2O vapor favored the growth of $MoSe_2$ domains, while transitioning to Ar+H_2 (5%) promoted the growth of WSe_2. This growth mechanism relied on the selective control of the carrier gas environment. The presence of oxidizing gases, mainly H_2O vapor, facilitated the evaporation of Mo-related precursors due to their higher oxidation rate than W-related precursors. Subsequently, these Mo-related precursors were deposited on the substrate at lower temperatures, acting as initial nucleation sites, followed by forming Mo-related crystalline domains. A swift change in the carrier gas from N_2+H_2O to Ar+H_2 rapidly depleted the supply of Mo precursors due to their high reduction rate while maintaining the supply of W precursors. This vapor-phase modulation of oxide species was critical in driving the sequential growth of multi-junction lateral heterostructures.

Photoluminescence Measurements

EHP PL imaging experiments were performed by using an optical parametric oscillator (OPO) system (APE picoEmerald) tunable from 750 to 950 nm, with 7 picosecond pulse width, and 80 MHz repetition rate. The PL imaging was performed by scanning the laser with a set of galvanometric mirrors (LaVison BioTec) in a Nikon microscope. The laser beam was focused on the sample by a 40x objective with numerical aperture N.A. = 0.95. The backscattered PL signal was collected by the same objective, reflected by a dichroic mirror that reflects below 690 nm, and then directed to a PMT. Distinct band pass filters were placed in front of the PMT in order to select the detected wavelength ranges. EHP PL images were made by an image acquisition software (LaVision BioTec Imspector Pro).

The PL intensities were analyzed using the ImageJ software.

EHP PL spectroscopy measurements were carried out using the same OPO laser and objective. The backscattered PL signal was directed to the spectrometer (*Andor* Shamrock 303i) equipped with a sensitive CCD camera (*Andor* IDUS DU401A-BV). Distinct short pass filters were placed before the spectrometer to block the reflected laser signal and collect only the higher energy PL emission. Conventional low density PL spectroscopy measurements were obtained in a confocal spectrometer (WITec Alpha 300R) using a 633 nm laser line and a 100× objective lens. The laser power was kept below 0.5 mW for all measurements.

Theoretical Calculations

The theoretical calculations in this study were performed using the SIESTA code [4], which implements Density Functional Theory (DFT) [5,6]. The Perdew–Burke–Ernzerhof functional (PBE) [7], within the Generalized Gradient Approximation (GGA), was employed for the treatment of exchange-correlation potential. In order to describe the interaction between valence electrons and the core states, non-local norm-conserving pseudopotentials, using the Troullier-Martins approximation [8], were applied. Specifically, for tungsten (W) atoms, a pseudopotential including relativistic effects, which are important for the treatment of heavy elements, was adopted. The basis set used was of the DZP type (Double-Zeta plus Polarization), with an energy deviation criterion of 0.01 Ry. The energy cutoff applied in all calculations was 300 Ry, with interatomic forces limited to 0.04 eV/Å and a convergence criterion for the density matrix of 10^{-5}.

The interface between the $MoSe_2$ and WSe_2 domains in the lateral heterostructure we employ in our calculations has an alloyed composition and extends over $\sim$3 nm (on the scale of the interfaces in our samples). The structural optimization was performed by

relaxing only the ionic positions. A uniform Monkhorst-Pack k-point grid with a density of 6 x 6 x 1 was used. The unit cell was constructed with the lattice parameters a = 20.05 Å and b = 92.68 Å, while the perpendicular direction c a vacuum of 15 Å was applied.

The absorption spectrum in Figure 2a was also obtained using the SIESTA code, based on the first-order perturbation theory formalism from which we obtain the imaginary part of the dielectric function as a function of energy. All calculations were performed for linearly polarized light, with the electric field direction along (1, 0, 0). Since it is well known that DFT tends to underestimate the electronic bandgap in materials and the non-interacting dielectric function often underestimates the HOMO–LUMO gaps, we use a scissor-operator shift of 0.32 eV for the lateral heterostructures, in order to shift up the unoccupied bands and to correct this effect in the optical spectrum, with the experimental bandgap of the $Mo_{0.5}W_{0.5}Se_2$ alloy (1.58 eV)[9–11] as a reference. A dense k-point grid of (8 x 4 x 1) was used to sample the different optical transitions along the Brillouin zone.

Section S2. Carrier Densities Calculation

The charge carrier density n_0 (cm^{-2}) generated under a pulsed excitation was calculated by:[12,13]

$$n_0 = \frac{P.\sigma}{A.f_{rep}.E}, \tag{1}$$

in which P is the incident laser power (W), σ is the absorptance taken from Yiley *et al.*[14], A is the area (cm^2) of the incident laser spot at the sample, f_{rep} is the laser pulse repetition rate (s^{-1}), and E is the incident photon energy (J). Note that the pump fluence used in the manuscript is the ratio between P and the product of A with f_{rep}, being A in m^2 in this case.

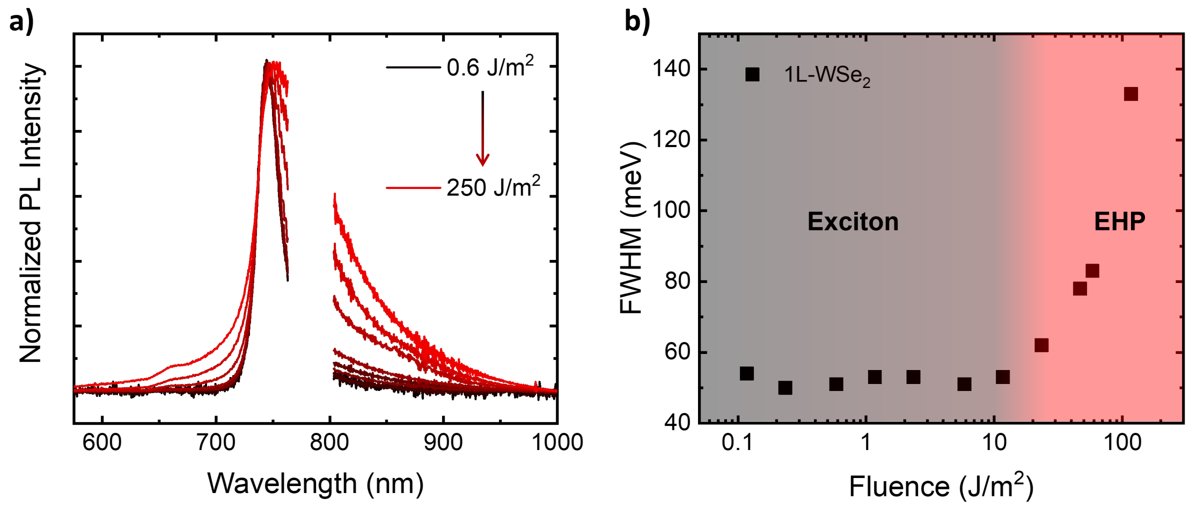

Figure S1: **a** Normalized PL spectra of a 1L-WSe_2 obtained at a 785 nm excitation and for different pump fluences. The observed broadening indicates the ionization of excitons in an EHP. **b** Power dependence of the 1L-WSe_2 PL full width at half maximum (FWHM). Below 10 J/m^2, the constant PL FWHM points to the excitonic regime, while the increasing FWHM above 10 J/m^2 denotes the EHP formation.

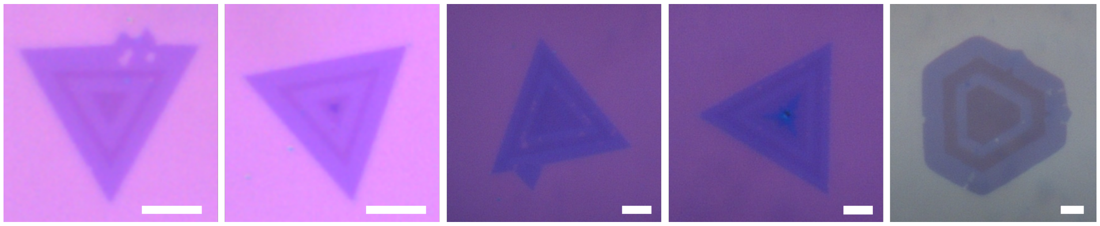

Figure S2: Optical images of distinct 1L and a 2L (last one) $MoSe_2$-WSe_2 lateral heterostructures studied in this work. The alternation of $MoSe_2$ and WSe_2 domains from the center to the edge can be observed, in which $MoSe_2$ regions are darker with respect to WSe_2 regions. Scale bars: 5 μm.

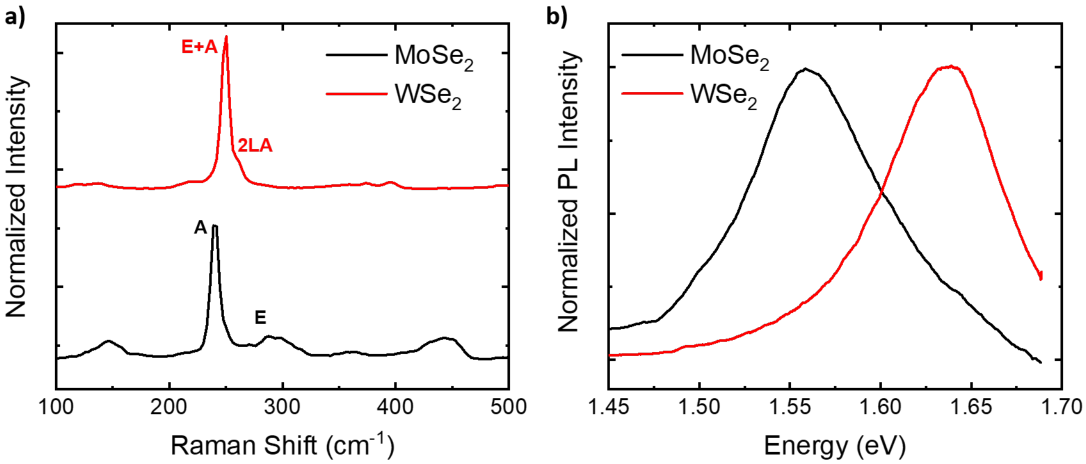

Figure S3: Raman and Photoluminescence (PL) characterization of the 1L-$MoSe_2$-WSe_2 lateral heterostructure. **a** Raman spectra of $MoSe_2$ (in black) and WSe_2 (in red) regions. The $MoSe_2$ Raman spectrum presents A_{1g} and E^1_{2g} modes at 241 and 281 cm^{-1}, respectively, while the WSe_2 displays its degenerated A_{1g} and E^1_{2g} modes at 249 cm^{-1} and 2LA mode at 257 cm^{-1}. **b** Normalized PL spectra of $MoSe_2$ (in black) and WSe_2 (in red) regions. Their single PL peaks are located at 1.56 and 1.64 eV for $MoSe_2$ and WSe_2, respectively. The Raman and PL spectra confirm the 1L thickness of both $MoSe_2$ and WSe_2 regions of this CVD grown sample[1–3]. Raman (PL) spectra were taken in a confocal spectrometer (WITec Alpha 300R) using a 532 (633) nm laser line and a 100× objective lens. The laser power was kept below 0.5 mW during all measurements.

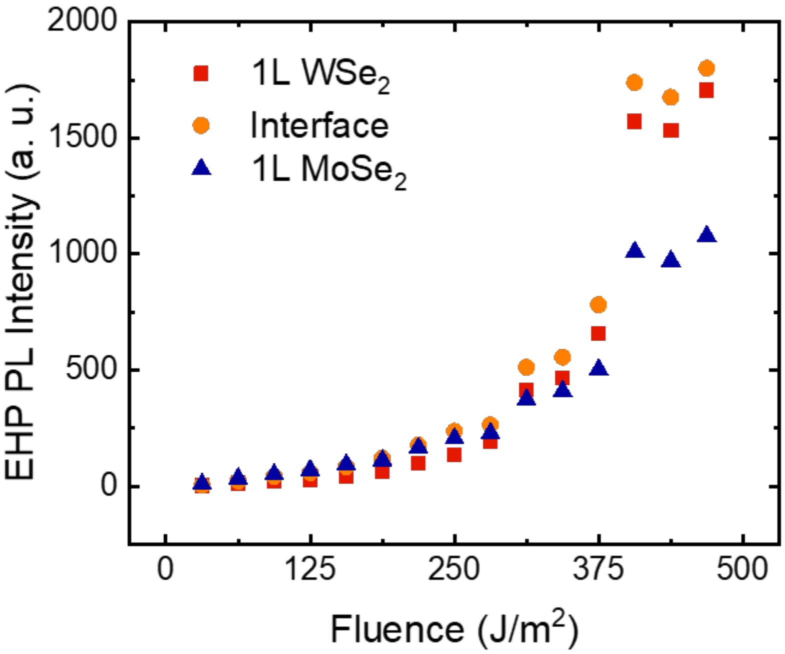

Figure S4: Power dependence of the PL intensity for $MoSe_2$, WSe_2 and interface regions of a 1L-$MoSe_2$-WSe_2 lateral heterostructure. The intensities were extracted from PL imaging experiments performed with a 600 J/m^2 pump fluence and collecting the signal with 690 nm short pass and 560 nm long pass filters in front of the PMT. Therefore, the exciton emission was blocked and only the high energy tail of the EHP signal was collected (see Figure S1). Besides, the PL intensities exhibit a nonlinear power dependence, further confirming the EHP regime.

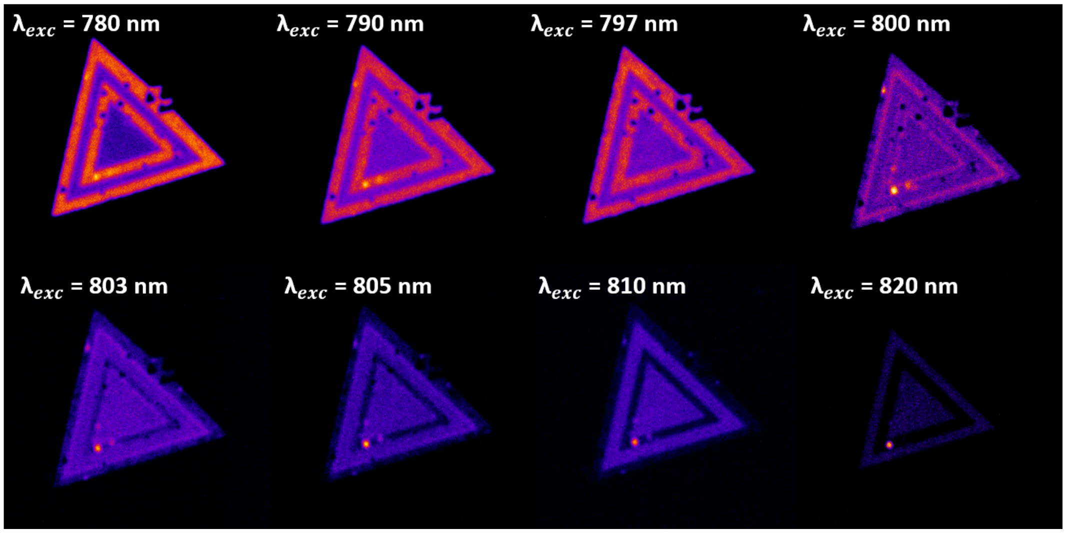

Figure S5: EHP PL imaging of a 1L-$MoSe_2$-WSe_2 lateral heterostructure for several excitation wavelengths, a 600 J/m^2 pump fluence and collecting the PL signal with a 620/60 nm band pass filter in front of the PMT. This 1L sample is the same as presented in the manuscript. However, here the excitation wavelength in which the enhanced emission at the heterojunction is maximum is 800 nm due to the distinct incident power.

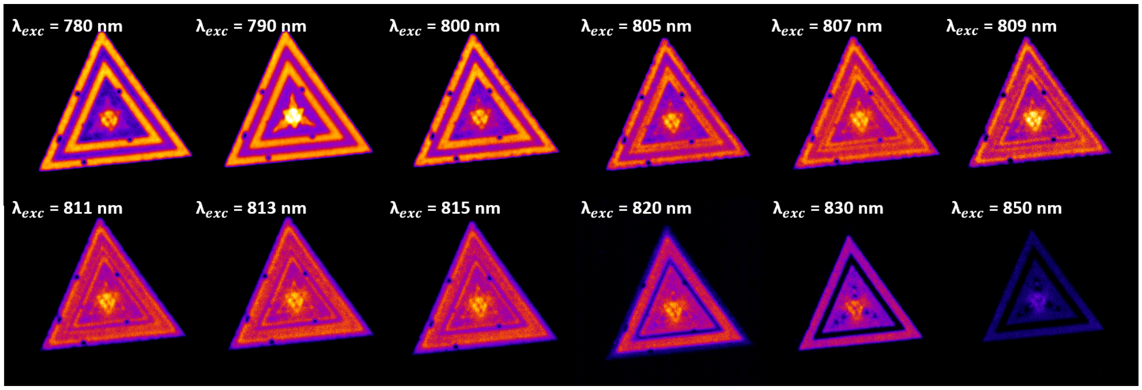

Figure S6: EHP PL imaging of a 1L-$MoSe_2$-WSe_2 lateral heterostructure for several excitation wavelengths, a 600 J/m^2 pump fluence and collecting the PL signal with 690 nm short pass and 560 nm long pass filters in front of the PMT. This 1L sample is not the same as the one presented in the manuscript. Although the pumping power used to probe it was the same used in the mappings of Figure S5, here the excitation wavelength in which the enhanced emission at the heterojunction is maximum is 809 nm due to the distinct wavelength range of detection.

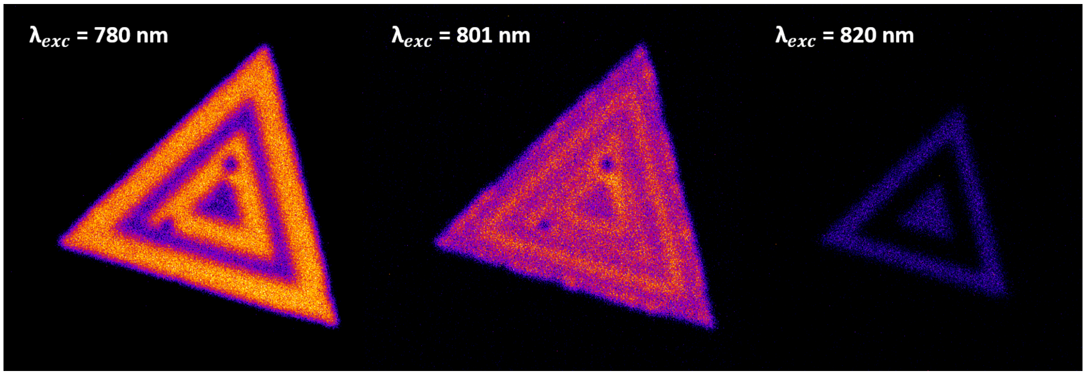

Figure S7: EHP PL imaging of a 1L-$MoSe_2$-WSe_2 lateral heterostructure for different excitation wavelengths, a 600 J/m^2 pump fluence and collecting the PL signal with a 620/60 nm band pass filter in front of the PMT. This 1L sample is not the same as the one presented in the manuscript. For these scanning measurements, the excitation wavelength in which the enhanced emission at the heterojunction is maximum is 801 nm, similar to the observed in Figure S5.

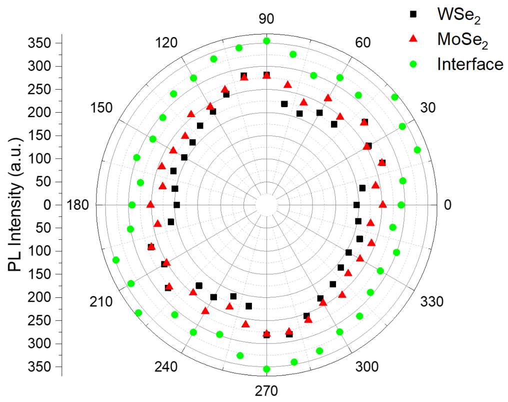

Figure S8: Polarization-resolved EHP PL measurement of the 1L-$MoSe_2$-WSe_2 lateral heterostructure. The graph displays polarized plots of both domains and from their interface for a 804 nm excitation laser wavelength and 600 J/m^2 pump fluence. No polarization dependence was observed for the EHP PL emission, discarding a coherent superposition interference as the reason of the heterojunction enhanced signal[15].

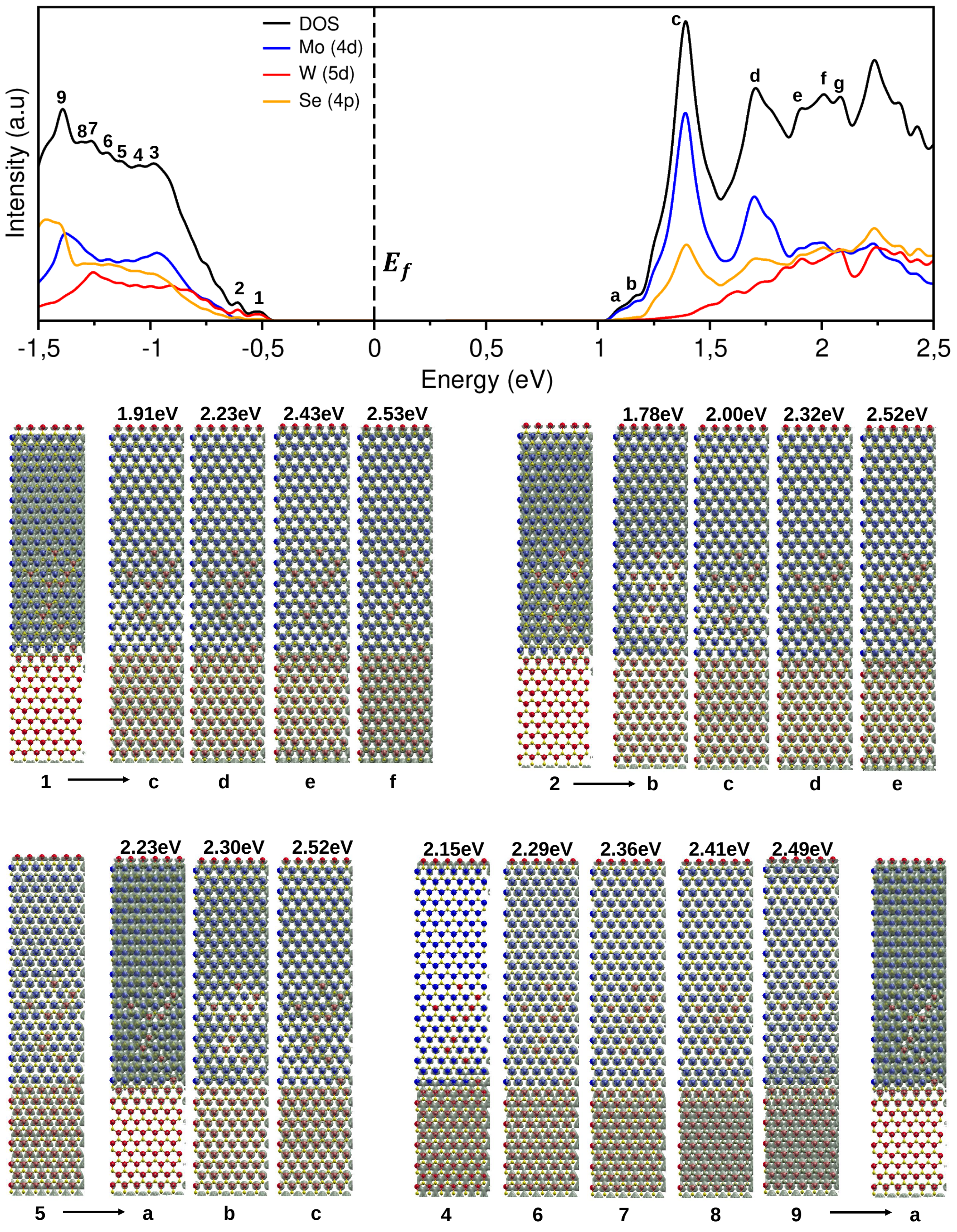

Figure S9: Top panel: total (DOS) and projected (PDOS) density of states of the $MoSe_2$-WSe_2 heterostructure. Bottom panels: initial and final states of selected optical transitions in our calculations. Peaks below the Fermi level (E_F), labeled with numbers, correspond to the initial states, while peaks above E_F, labeled with letters, correspond to the final states. Isosurfaces of the local density of states (LDOS) are shown for selected transitions, highlighting the wavefunction overlap at the $MoSe_2$-WSe_2 interface. Transition energies are indicated above the corresponding LDOS images.

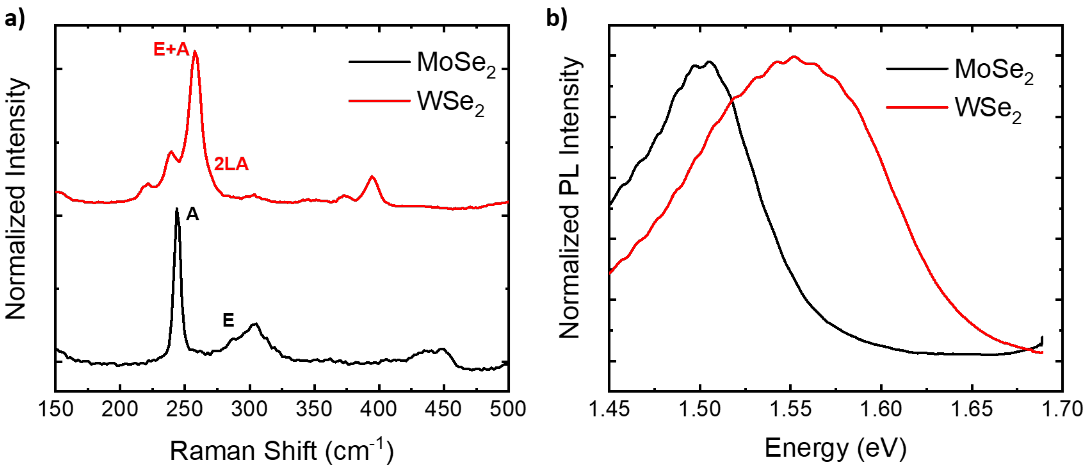

Figure S10: Raman and Photoluminescence (PL) characterization of the 2L-$MoSe_2$-WSe_2 lateral heterostructure. **a** Raman spectra of $MoSe_2$ (in black) and WSe_2 (in red) regions. The $MoSe_2$ Raman spectrum presents A_{1g} and E^1_{2g} modes at 243 and 285 cm^{-1}, respectively, while the WSe_2 displays its degenerated A_{1g} and E^1_{2g} modes at 255 cm^{-1} and 2LA mode at 263 cm^{-1}. **b** Normalized PL spectra of $MoSe_2$ (in black) and WSe_2 (in red) regions. Their single PL peaks are located at 1.50 and 1.55 eV for $MoSe_2$ and WSe_2, respectively. The Raman and PL spectra confirm the 2L thickness of both $MoSe_2$ and WSe_2 regions of this CVD grown sample[1–3]. Raman (PL) spectra were taken in a confocal spectrometer (WITec Alpha 300R) using a 532 (633) nm laser line and a 100× objective lens. The laser power was kept at 0.3 mW during all measurements.

References

[1] Sahoo, P. K.; Memaran, S.; Xin, Y.; Balicas, L.; Gutiérrez, H. R. One-pot growth of two-dimensional lateral heterostructures via sequential edge-epitaxy. *Nature* **2018**, *553*, 63–67.

[2] Sahoo, P. K.; Memaran, S.; Nugera, F. A.; Xin, Y.; Díaz Márquez, T.; Lu, Z.; Zheng, W.; Zhigadlo, N. D.; Smirnov, D.; Balicas, L.; Gutiérrez, H. R. Bilayer Lateral Heterostructures of Transition-Metal Dichalcogenides and Their Optoelectronic Response. *ACS Nano* **2019**, *13*, 12372–12384.

[3] Nugera, F. A.; Sahoo, P. K.; Xin, Y.; Ambardar, S.; Voronine, D. V.; Kim, U. J.; Han, Y.; Son, H.; Gutiérrez, H. R. Bandgap Engineering in 2D Lateral Heterostructures of Transition Metal Dichalcogenides via Controlled Alloying. *Small* **2022**, *18*, 2106600.

[4] Soler, J. M.; Artacho, E.; Gale, J. D.; García, A.; Junquera, J.; Ordejón, P.; Sánchez-Portal, D. The SIESTA method for ab initio order-N materials simulation. *Journal of Physics: Condensed Matter* **2002**, *14*, 2745.

[5] Hohenberg, P.; Kohn, W. Inhomogeneous Electron Gas. *Phys. Rev.* **1964**, *136*, B864–B871.

[6] Kohn, W.; Sham, L. J. Self-Consistent Equations Including Exchange and Correlation Effects. *Phys. Rev.* **1965**, *140*, A1133–A1138.

[7] Perdew, J. P.; Burke, K.; Ernzerhof, M. Generalized Gradient Approximation Made Simple. *Phys. Rev. Lett.* **1996**, *77*, 3865–3868.

[8] Troullier, N.; Martins, J. L. Efficient pseudopotentials for plane-wave calculations. *Phys. Rev. B* **1991**, *43*, 1993–2006.

[9] Tongay, S.; Narang, D. S.; Kang, J.; Fan, W.; Ko, C.; Luce, A. V.; Wang, K. X.; Suh, J.; Patel, K. D.; Pathak, V. M.; Li, J.; Wu, J. Two-dimensional semiconductor alloys: Monolayer Mo1xWxSe2. *Applied Physics Letters* **2014**, *104*, 012101.

[10] Afrid, S. M. T.-S.; Utsha, S. G.; Zubair, A. First-principles study on tunable optoelectronic properties of monolayer Mo1xWxSe2 alloys and defect engineered electronic properties of Mo1xWxSe2 alloys. *Physica Scripta* **2023**, *98*, 104002.

[11] Zhang, M.; Wu, J.; Zhu, Y.; Dumcenco, D. O.; Hong, J.; Mao, N.; Deng, S.; Chen, Y.; Yang, Y.; Jin, C.; Chaki, S. H.; Huang, Y.-S.; Zhang, J.; Xie, L. Two-Dimensional Molybdenum Tungsten Diselenide Alloys: Photoluminescence, Raman Scattering, and Electrical Transport. *ACS Nano* **2014**, *8*, 7130–7137, PMID: 24884059.

[12] Jue, W.; Jenny, A.; Yusong, B.; Alexander, S.; Matthias, F.; Frank, J.; Xiaodong, X.; Mackillo, K.; James, H.; X.-Y., Z. Optical Generation of High Carrier Densities in 2D Semiconductor Heterobilayers. *Science Advances* **2019**, *5*, eaax0145.

[13] Sousa, F. B.; Perea-Causin, R.; Hartmann, S.; Lafetá, L.; Rosa, B.; Brem, S.; Palekar, C.; Reitzenstein, S.; Hartschuh, A.; Malic, E.; Malard, L. M. Ultrafast hot electron–hole plasma photoluminescence in two-dimensional semiconductors. *Nanoscale* **2023**, *15*, 7154–7163.

[14] Li, Y.; Chernikov, A.; Zhang, X.; Rigosi, A.; Hill, H. M.; Van Der Zande, A. M.; Chenet, D. A.; Shih, E.-M.; Hone, J.; Heinz, T. F. Measurement of the optical dielectric function of monolayer transition-metal dichalcogenides: MoS 2, Mo S e 2, WS 2, and WS e 2. *Physical Review B* **2014**, *90*, 205422.

[15] Sousa, F. B.; Lafeta, L.; Cadore, A. R.; Sahoo, P. K.; Malard, L. M. Revealing atomically sharp interfaces of two-dimensional lateral heterostructures by second harmonic generation. *2D Materials* **2021**, *8*, 35051.
